# Silk Fibroin-Based Piezoelectric Sensor with Carbon Nanofibers for Wearable Health Monitoring Applications

**DOI:** 10.3390/s23031373

**Published:** 2023-01-26

**Authors:** Senthil Kumar Rathinasamy, Rajagopal Maheswar, Josip Lorincz

**Affiliations:** 1 Department of Mechanical Engineering, KPR Institute of Engineering and Technology, Coimbatore 641407, India; 2 Department of ECE, Centre for IoT and AI (CITI), KPR Institute of Engineering and Technology, Coimbatore 641407, India; 3 Faculty of Electrical Engineering, Mechanical Engineering and Naval Architecture (FESB), University of Split, 21000 Split, Croatia

**Keywords:** piezoelectricity, carbon nanofiber, polymers, biomedical, wearable devices, healthcare, real-time monitoring, signals

## Abstract

The continuous real-time monitoring of human health using biomedical sensing devices has recently become a promising approach to the realization of distant health monitoring. In this paper, the piezoelectric characteristics of the silk fibroin (SF) natural polymer were analyzed as the material used for obtaining sensing information in the application of distance health monitoring. To enhance the SF piezoelectricity, this paper presents the development of a novel SF-based sensor realized by combining SF with different carbon nanofiber (CNF) densities, and for such newly developed SF-based sensors comprehensive performance analyses have been performed. Versatile methods including the scanning electron microscope, Fourier transform infrared spectroscopy, Raman and X-ray diffraction measurements and impedance analysis were used to study the morphologic, mechanical and electrical properties of the developed SF-based sensor. The SF with CNF samples was analyzed for three different pressure loads (40 N, 60 N and 80 N) in 500 compression test cycles. The analyses thoroughly describe how combining natural polymer SF with different CNF densities impacts the piezoelectricity and mechanical strength of the proposed SF-based sensor. The developed piezoelectric SF-based sensors were further tested on humans in real medical applications to detect generated piezoelectric voltage in versatile body movements. The maximum piezoelectricity equal to 2.95 ± 0.03 V was achieved for the jumping movement, and the SF sample with a CNF density equal to 0.4% was tested. Obtained results also show that the proposed SF-based sensor has an appropriate piezoelectric sensitivity for each of the analyzed body movement types, and that the proposed SF-based sensor can be applied in real medical applications as a biomedical sensing device. The proposed SF-based sensor’s practical implementation is further confirmed by the results of cytotoxicity analyses, which show that the developed sensor has a non-toxic and biocompatible nature and can be efficiently used in skin contact for biomedical wearable health monitoring applications.

## 1. Introduction

In recent years, demand for bioinspired smart wearable sensors which will be implemented in biomedical and healthcare monitoring devices has gained momentum. Injuries, degenerative diseases, musculoskeletal disorders and neurological impairments frequently result in impaired walking and motility. In the past, medical professionals used physical and medical examinations to diagnose these issues. Only a small proportion of patients can undergo complete gait analysis, which is only possible at larger tertiary hospitals. Many scientists advocated that all patients with degenerative disorders and those in need of long-term rehabilitation should undergo gait analysis that requires the use of human gait monitoring instruments [1]. Due to the lack of proper instruments, data related to monitoring gait disabilities and patient motility are difficult to collect for clinicians and hospitals [2]. In this work, the main aim is to develop a sensor for analyzing human locomotion. These sensors can be used for motion studies and can be exploited in the development of gait-measuring instruments.

Gait analysis is the systematic examination of human locomotion. In this type of analysis, quantities that define human movement are measured, described and evaluated. Gait analysis allows for the identification of the gait phase, the determination of the kinematic and kinetic parameters of human gait events, and the quantitative assessment of musculoskeletal functioning [3]. Therefore, gait analysis has been used in sports, rehabilitation and medical diagnoses. For instance, in some sports training the technique is used to identify performance flaws in athletes in order to improve their locomotion and consequently sports results [4]. Gait analysis is also used in orthopedics and rehabilitation to track a patient’s recovery. Accelerometers and gyroscopes have been studied for use in health diagnostics to distinguish between asymptomatic persons and patients with medial knee osteoarthritis. Additionally, gait analyses are used in ambulatory monitoring as an application for monitoring Parkinson’s disease [5]. Gait analysis has been a key technique and helpful instrument in the field of biomedical engineering to describe human locomotion. The study presented in [6] indicates strong researchers’ and physicians’ interest in gait analysis.

Gait analysis has been studied since the late-19th century, and with the advent of video camera systems it has found widespread use in biomedical engineering. A common gait analysis technique has been created and used effectively in a variety of gait laboratories [7]. It is based on a multi-camera motion capture system and force platform with the ability to measure ground reaction forces caused by body movements. However, implementation of such common gait analysis needs specialized locomotion laboratories, expensive sensing devices, complex measuring setup and long data post-processing analyses [8]. In addition, restrictions in terms of the patient’s moving area and gait cycles have been noted. Due to all these reasons there is a need for an alternative gait analysis technique based on wearable biosensors, which are less expensive and may be used outside of the restricted size laboratories [6].

Polymers have been widely researched in recent years as materials for developing biosensors in wearable applications, due to their chemical, physical, mechanical and biological properties. For that reason, the piezoelectric sensors for wearable electronics are mostly made from a variety of synthetic polymers. Due to their potential use in health care, biosensors have recently gained a lot of attention in the field of wearable electronics. The main characteristic that is exploited in the implementation of such biosensors is the effect of piezoelectricity. Piezoelectricity is the ability of certain materials to generate voltage because of the electric charge that is accumulated in some solid materials as a response to applied vibration or mechanical stress. Piezoelectricity can be found in ceramic materials like barium titanate (BaTiO3) [9] and lead zirconate titanate (PZT) [10], as well as in synthetic polymers such as polyvinylidene fluoride (PVDF) and its copolymers [11]. In recent years, biopolymers such as Polylactic acid (PLA) [12] or gelatin [13] have been used for achieving intrinsic shear piezoelectricity, where the electrical output is obtained through mechanical pressure. Nevertheless, due to its good piezoelectric properties silk fibroin (SF) as a natural and biocompatible polymer has also emerged in many biomedical applications which exploit the piezoelectric sensing concept [14].

The SF is a natural protein that originates from the silkworm (Bombyx mori), which is a natural, eco-friendly and biodegradable biopolymer. The SF contains glycine, alanine, serine amino acids, acetylated terminal ser residues, and above all, intrinsic shear piezoelectricity [15,16]. The SF is hydrophobic, biocompatible and controlled biodegradable with high mechanical strength and flexibility, and it is well suited for high-strength applications [17]. The SF combined in composites does have high mechanical stretching and flexibility [15]. The silk cocoons contain two proteins: fibroin and sericin [18]. Sericin can be removed by degumming processes using high temperature and pressure, enzymes, acids, or alkalines [19]. After the degumming process of sericin, pure fibroin is left, which can be dissolved to get structures in the semicrystalline form [17,18,19,20]. The SF itself is crystalline in nature whereas the Silk I crystals are orthorhombic, while the Silk II crystals have monoclinic unit cells [21,22].

Besides the emerging application of SF in the field of biosensing, the possibilities of versatile implementation of carbon nanofibers (CNFs) gain momentum. The CNFs are nanostructures with graphene layers arranged in a cylindrical or conical shape, with lengths ranging from sub-microns to millimeters and diameters spanning from a few to hundreds of nanometers. The CNFs are conductors having electric properties similar to those of graphite. The CNFs show notable implementation potential in different areas, which span tissue engineering, catalysis, environmental science, materials science, biomedicine and energy. Initial tests of the performance of the SF with CNFs as combined materials have been performed by Su et al. in [23] for establishing triboelectric nanogenerators. The electric signal generation was obtained in the amount of 140.99 µW/cm^2^ in the testing instrument and 317.4 µW/cm^2^ with hand tapping, having sensors positioned on human fingers [23].

Therefore, the initial testing results show that combining SF with CNFs can have potential implementation as biosensors in the area of biomedical and healthcare applications. The main goal of this paper is to test these hypotheses and to show the operational capabilities of piezoelectric sensing of such materials realized by combining the SF with CNFs. Another goal of this paper is to show what are the possibilities for practical implementation of such sensing material in health monitoring applications. Hence, the following are the main contributions of this work:

- Description of the development process of a novel biodegradable sensor based on the combination of the SF with different densities of CNFs;
- Presentation of the analyses of the piezoelectricity voltage levels that the proposed SF-based biosensor can generate with respect to the different CNF densities;
- Presentation of the analyses on the mechanical response of a developed SF-based sensor for different cyclic compressions;
- Presentation of the analyses of the proposed SF-based biosensor performance in the case of its practical implementation on real humans during different human motion activities.

According to the contributions listed above, the proposed article has been organized into the four remaining sections: In Section 2, related work dedicated to the techniques for the development and analyses of materials based on the SF and CNF is presented. Section 3 elaborates on the technical details of the development process of the proposed SF-based sensor with different CNF densities. Performance analyses of the proposed SF-based sensor in terms of mechanical, electrical, implementation and cytotoxicity characteristics are discussed in detail in Section 4. Finally, Section 5 summarizes the overall contribution of the paper and discusses future research directions.

## 2. Literature Review

Liu et al. developed a green-based SF mat self-powered pressure sensor. This sensor produces 30.6 mV/N and has a fast response equal to 3.4 ms with good durability [24]. The sensor is used in oral medicine devices and in vivo pressure monitoring. Kim et al. developed SF-based biodegradable composite generators for implantable devices [25]. The piezoelectric output was equal to 1.8 V and 0.1 µA/cm^2^ in footstep. When compared to the levels of piezoelectricity obtained in this work, in both works [24,25] the generated SF voltages are lower.

An environmentally friendly SF bio-electric generator was developed by Kim et al. in work [26]. The electrospinning technique with polyimide is used to produce the SF. A capacitor’s ability to be swiftly charged up to 2 V has been demonstrated using the designed bioelectric generator. Aqueous air drying based on transforming the SF substrate into films and marking the silk films to the required draw ratio were the two steps used by Yucel et al. in the development of silk films [15]. Zone drawing and water immersion were researched as the two distinct methods for preparing silk materials for piezoelectric experiments. With the analyzed samples, authors in work [15] attained a piezoelectric voltage of 2.7 V. An energy-harvesting spider SF-based piezoelectric nanogenerator was developed by Maiti et al. in work [27]. According to the presented results, it is possible to get an enhanced output voltage of 21.3 V and current of 0.68 A which has an impactful power density of 4.56 W/cm^2^. Additionally, in work [27] the authors create a potent spider silk-based piezoelectric nanogenerator that exhibits high efficiency (66%), high output voltage (21.4 V) and current (0.69 A), as well as an immediate power density of roughly 4.57 W/cm^2^.

In [28] it is shown that the CNFs in very low concentration successfully improved the piezoelectricity of PVDF when deployed as piezoelectric sensors [28]. This motivates testing of the main hypothesis in this paper, which is related to the idea that a similar approach for the SF can be considered in terms of investigating how different concentrations of CNFs in the SF can result in the development of the biodegradable and implantable piezoelectric sensor. Additionally, in the presented overview of published related works, it is shown that low piezoelectric voltage generation has been achieved and an investigation on the possibilities of achieving higher piezoelectric voltage generation for materials that combines SF with different concentrations of the CNF is missing. Therefore, in this study the natural polymer SF was used in combination with acid-functionalized CNFs having various concentration densities (0.1%–0.5% (*w*/*v*)). The prepared materials were categorized for investigation into their size, shape, structure (morphology), crystal analysis, functional groups and mechanical and electrical attributes. Moreover, in the paper the performance of the developed sensor was studied in terms of the compressive load, cyclic continuous load, wash durability and toxicity. The performance of the developed piezoelectric sensor is tested with male and female volunteers wearing sensors in real applications. The wireless Bluetooth technology was implemented to transfer data and LabVIEW is the software platform used for application data storage and analyses.

## 3. Materials and Methods Used for Biosensors Development

### 3.1. Preparation of Silk Fibroin

The SF used in the analyses has been firstly chemically prepared for usage as the bioelectric sensor. For the preparation of the SF the chemicals and solvents were procured from Himedia laboratories Pvt. Ltd. Mumbai, India. Bombyx mori silk cocoons (TANSILK, Coimbatore, India) were degummed using the 0.5 M of sodium carbonate (Na_2_CO_3_) application at 90 °C for 1.5 h and the SF material was rinsed three times with deionized (DI) water to remove the sericin. The degummed SF material was dissolved in Ajisawa’s reagent (Calcium Chloride (CaCl_2_)/ethanol (CH_3_CH_2_OH)/DI water) in a molar ratio of 1:2:8 at 50 °C until pure substrate was obtained (the total process lasts 4 h). The prepared substrate was dialyzed in distilled water for 3 days at 15 °C and again followed with dialysis against polyethylene glycol (PEG) for an additional 24 hours to remove salt and to concentrate the pure SF substrate. The prepared SF substrate was dried and kept at 5 °C for further use.

### 3.2. Synthesis of Acid-Functionalized CNFs

In the process of developing a new biosensor the preparation of the CNFs for synthesis has been initially performed. To achieve the CNF functionalization, the 5 g of the CNF (100 nm diameter, 20–100 µm length, manufacturer Sigma-Aldrich, St. Louis, MO, USA) was mixed with the 100 mL of the concentrated nitric acid (HNO_3_, 70% conc.) and sulfuric acid (H_2_SO_4_, 98% conc.) in a 1:3 ratio (protocol as in [29]). After 4 h it was diluted with water and filtered using 0.2 µm acetate paper. The resulting CNF was dried for 60 h at 40 °C.

### 3.3. Preparation of SF with CNFs Nanocomposite Sponges

The 5 mL of the SF substrate was mixed with the 250 µL of glutaraldehyde at 75 °C. For the preparations of nanocomposite sponges solvent-assisted methods were followed. In parallel, previously prepared acid-functionalized CNFs in densities ranging between 0.1% and 0.5% (*w*/*v*) powder were mixed with the SF substrates. Furthermore, two hours of stirring and 40 min of ultrasonic treatment were done to get a proper dispersion of the CNF in the SF substrates. Then the solvent was preserved in a deep freezer for 24 h. After freezing the samples were freeze-dried in a lyophilizer (Alpha 2-4 LD plus model, manufacturer Martin Christ, Osterode am Harz, Germany) and a sponge was obtained after 7 h. After the finalization of this process, lyophilized nanocomposite sponges the SF and SF with CNF sponges were used in further performance studies.

### 3.4. Characterization of Material Combining SF and CNF Nanocomposites

**Figure 1:**
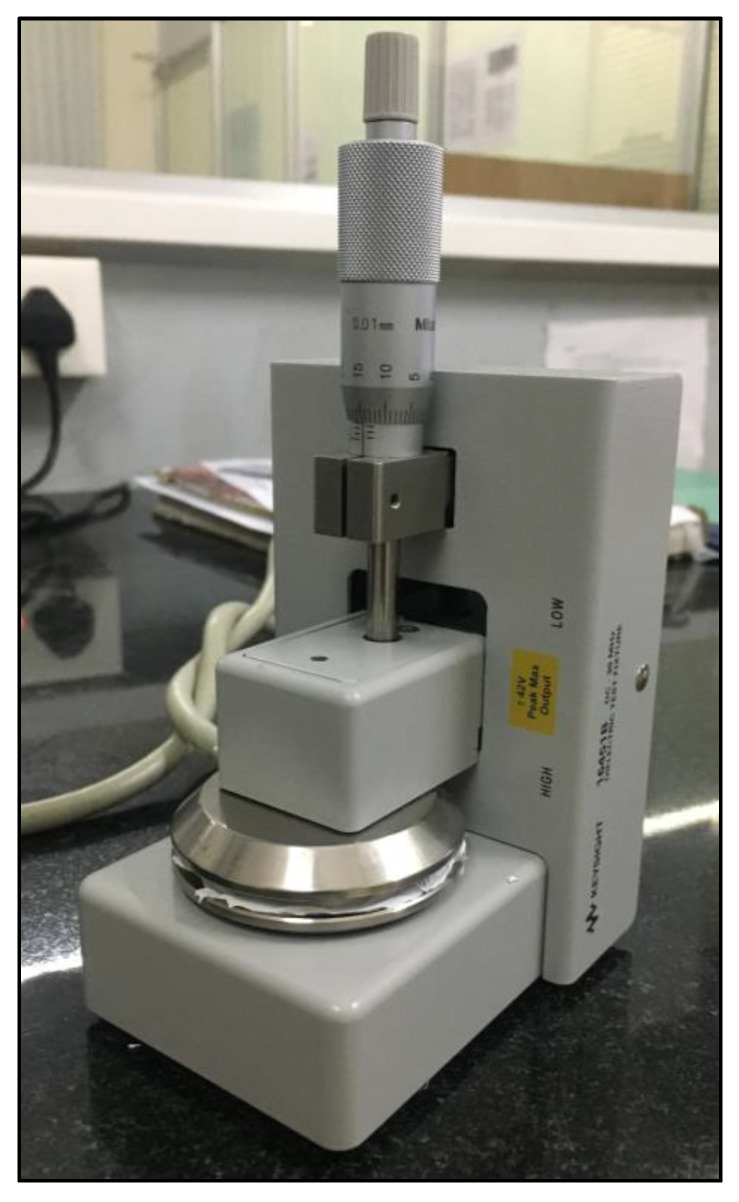
Probe measuring instrument (impedance analyzer).

The morphology of the pure SF and SF with CNF nanocomposite sponges was examined using scanning electron microscopy (SEM, manufacturer Carl Zeiss AG, EVO 18, Jena, Germany) at 10 kV after performing gold layer coating of the substrate which lasts 120 s. The porosity of the samples was calculated using the liquid displacement method [30]. The Fourier transform infrared (FTIR) spectroscopy was used to characterize the functional groups of nanocomposite samples (FTIR model 400 cm–4000 cm^−1^ range, IR affinity 1S series, manufacturer Shimadzu, Kyoto, Japan). A diffractometer (Empyrean platform, Cu Ka radiation 1.54, manufacturer Malvern Panalytical, Malvern, UK) was used for the X-ray diffraction (XRD) studies. Sponge thickness was measured by using the digital micrometer (manufacturer Mitutoyo, Kanagawa, Japan). The conductivity studies for the nanocomposite sponges were carried out in the impedance analyzer (manufacturer Keysight Technologies, Santa Rosa, CA, USA) connected with a dielectric test fixture. The atomic force microscope (AFM) was used to examine the nanofibers and the conducting Scanning Kelvin Probe Microscopy (SKM, manufacturer NT-MDT LLC, Moscow, Russia) was used for the AFM characterization in the non-contact mode with silicon nitride cantilever beam. To analyze the impedance of the developed nanosponge material, the circular shape SF sponge (samples) with the 12 mm Φ was placed in between the impedance probe setup presented in Figure 1.

The samples were tested in the frequency range between 20 Hz and 20 MHz. The compression studies (performed on model Instron 3366 universal testing machine, manufacturer Instron Ltd., Norwood, MA, USA) were carried out at room temperature equal to 27 °C with 10 kN cell load at speed of 2 mm/min. The storage and loss moduli (based on ASTM d3039 standard) were studied using Modular Compact Rheometer (model MCR102, manufacturer Anton Paar GmbH, Graz, Austria) with Dynamic Mechanical Analysis (DMA) attachment. The sample’s thickness was measured and clamped in the Solid Rectangular Fixture (SRF).

The experiment was performed at 40 °C at the frequency range of 1 Hz–10 Hz with 2% torsion mode. The piezoelectric test of the developed samples (diameter 3.8 cm) was carried out at a cam machine (in-house developed [31] for 100 cycles at an offset angle of 20°). In the mechano–electrical study, the load vs. voltage response piezoelectric test was performed at 40 N, 60 N and 80 N for 500 cycles in Motorized Tension/Compression Test Stand force gauge (model ESM303, manufacturer MARK 10, Copiague, NY, USA) and voltage response was simultaneously collected through the digital storage oscilloscope (DSO, model DSO1012A, manufacturer Agilent Technologies, Santa Clara, CA, USA).

The wash performance on piezoelectricity was evaluated for materials combining the SF and CNF samples. The samples were sandwiched between the stainless steel mesh (400 µm) and then washed with the 2% sodium hydroxide (NaOH) and the 2% soap solution for the one-hour duration. Further, the samples were washed twice with water and dried under a vacuum overnight at 50 °C. This experiment was repeated for 10 cycles to analyze the wash performance of the sample and to have statistical relevance of analyses. The cytotoxicity assay (MTT) was carried out as per the protocol described in [29]. The scaffolds were placed in a tube containing the 1:10 diluted blood in the phosphate-buffered saline (PBS) for the in vitro hemolysis assay. For positive control, cells were treated with the red blood cell (RBC) lysis buffer. All of the materials used were gently mixed and incubated for a period of one hour in the water bath at a temperature of 37 °C. After another hour of incubation all tubes were centrifuged at 2200 rounds per minute (rpm) for five minutes and the supernatant was examined for hemoglobin estimation. The absorbance was measured at 545 nm and hemolysis was calculated after the material incubation. The results were compared with the 1:10 diluted blood without biomaterial (control).

### 3.5. Wireless Monitoring of Human Gait

**Figure 2:**
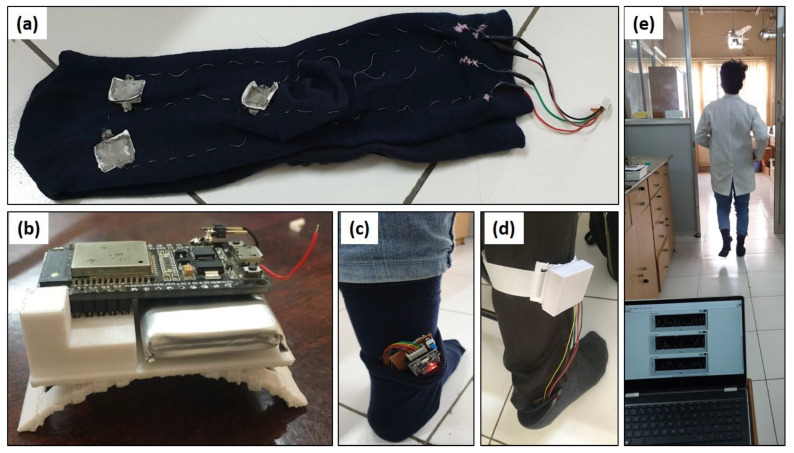
(**a**) The three SF-based sensors with CNF stitched in the socks by means of conducting thread; (**b**) Electronic controller unit; (**c**) Electronic controller unit connected with sensors; (**d**) Electronic controller unit covered with the 3D printed case; (**e**) Sensors testing with a volunteer and LabVIEW software [Video S1: movement analyses].

The Laboratory Virtual Instrument Engineering Workbench (LabVIEW VI, manufacturer National Instruments, Austin, TX, USA) is used as a system-design platform and development environment to display the collected data in a numerical and graphical representation (Figure 2d). The application of LabVIEW VI software v.2015 is primarily dedicated to monitoring the real-time signals from sensors via Bluetooth communication. Figure 2a shows testing socks and three SF sensors with CNF piezoelectric material. Testing socks are coupled with an electronic controller (ESP 32, manufacturer Espressif Systems, Shanghai, China) that is the low-power system on a chip microcontroller (Figure 2b,c) used to sense the foot pressure through the analog to digital conversion (ADC) port. The sensors were stitched on socks in 3 locations shown in Figure 2a (the toe, midsection and heel), using stainless steel conductive thread. The conductive thread helps to transfer the current and does not affect the human skin. Figure 2c,d shows that the microcontroller was covered up with the 3D-printed case for safety purposes. The volunteer testing with the developed sensors and sensing data collection through Lab view software is presented in Figure 2e [Video S1: movement analyses].

## 4. Results

### 4.1. Cross-Section Analysis

**Figure 3:**
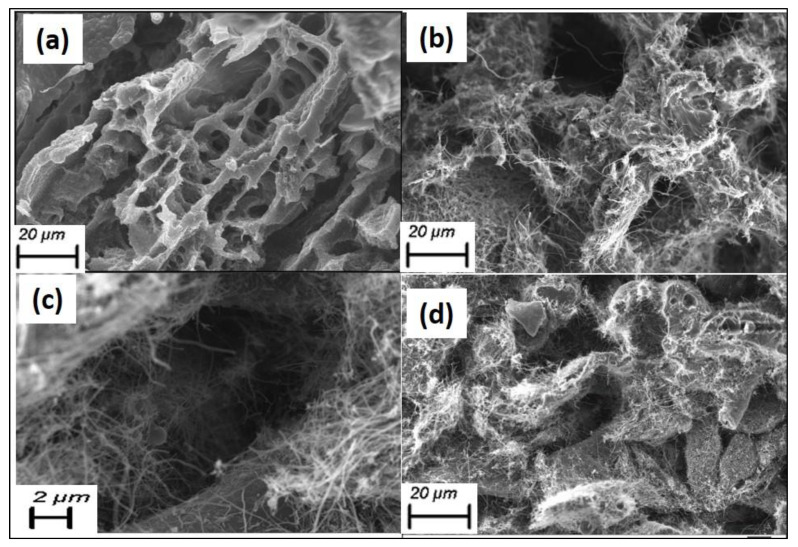
Morphological analysis of the SF and SF with CNF samples at (**a**) SF at 20 µm resolution; (**b**) SF with CNF density equal to 0.3%; (**c**) high magnification of SF with CNF density equal to 0.4% at 2 µm resolution; (**d**) SF with CNF density equal to 0.5% at 20 µm resolution.

The cross-section morphology analysis was performed for the pure SF samples and the SF with CNFs nanocomposite samples (Figure 3). In order to present the internal SF developments, Figure 3c shows the SF with CNF 0.4% sample with high magnification, respectively. According to results presented in Figure 3b–d, it can be seen that with the increment of CNFs concentration in the SF, the pores become lesser and the fibers were homogeneously dispersed in the overall cross-section area of the SF sponges. The CNFs, therefore, enriching the conducting network in the SF sample containing the CNFs. Figure 3b–d also shows that the CNFs are evenly dispersed in the SF matrix and they are connected between the layers of the SF. Results also show that for sample examination using the liquid displacement method, the pure SF sample and samples with nanocomposite sponges combined the SF with CNFs in the concentration of 0.1%, 0.2%, 0.3%, 0.4% and 0.5% have porosity percentages equal to 66 ± 2.23%, 57 ± 3.19%, 49 ± 3.64%, 41 ± 2.21%, 33 ± 3.74% and 27 ± 1.23%, respectively. Therefore, obtained results show that with the addition of the CNFs concentration the porosity of sponges becomes lower. Such uniform distribution of porous structures observed under the SEM can help to achieve compressive resiliency with reasonable strength to retain structural integrity.

### 4.2. XRD Analysis of Materials Combining the SF and CNFs

**Figure 4:**
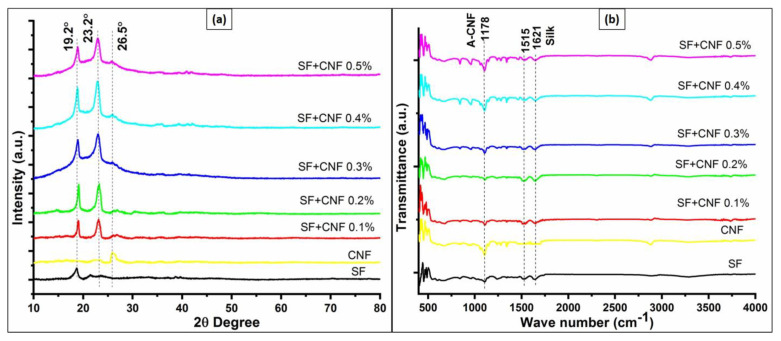
Crystal analysis of the SF sample and the SF sample with CNF nanocomposites in terms of: (**a**) XRD analyses; and (**b**) FTIR analysis.

The XRD patterns of the pure SF sample and the SF sample with the CNFs concentration equal to 0.1%, 0.2%, 0.3%, 0.4% and 0.5% in the sample are shown in Figure 4a. According to Figure 4a, the reason for detected SF XRD peaks is associated with the crystal structure of analyzed samples. The 2θ degree equal to the 19.2˚ diffraction peak is attributed to the Silk I crystal structure, and the 2θ degree equal to the 23.2° diffraction peak corresponds to the Silk II structure associated with crystalline beta-sheet [32]. The 2θ degree equal to the 26.5° diffraction peak corresponds to the CNFs, and it resembles the 002 lattice plane interlayer arrangement of carbon [33]. The crystallinities of the samples range from 46.4% to 60.1%. Moreover, according to Figure 4a it can be seen that with the increase in CNFs concentration the amount of crystal in Silk II is increasing in the composite. Since Silk II is responsible for the piezoelectricity of the silk these samples are expected to exhibit higher piezoelectricity. It has been observed that composite having the SF with the CNFs concentration of dispersed samples equal to 0.4% has the highest crystallinity. Beyond that level of CNFs concentration crystallinity starts to decrease, while smaller quantities of CNFs concentration have the opposite effect and they act as a nucleating site. Therefore, the increased CNF concentrations do not contribute to the improvement in the overall crystallinity of the SF.

Additionally, Figure 4b shows the occurrence of the SF and CNF in the analyzed FTIR spectra. The characteristic SF spectrum peaks at 1621 cm^−1^ correspond to amide I (C-O stretching) and the SF spectrum peaks at 1515 cm^−1^ correspond to amide II (N-H in-plane bending) [19]. Moreover, the characteristic peak at 1178 cm^−1^ shows the presence of the acid group (vibration of C-O) of the CNFs, and according to work [28] it can be superimposed with the carboxyl group of the silk.

**Figure 5:**
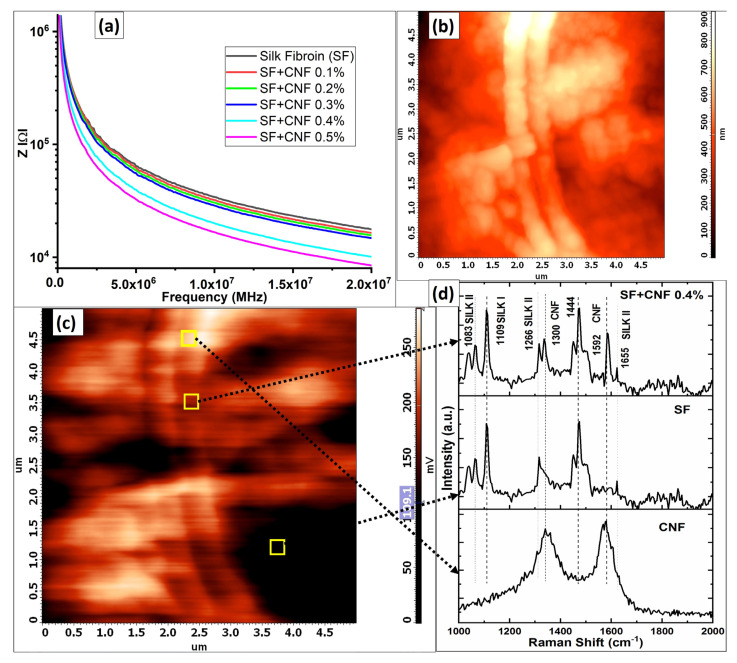
(**a**) Impedance vs. frequency graph for the SF sample having different CNF concentrations; (**b**) the AFM image of SF with CNFs network; (**c**) AFM image of SF with CNFs magnified in areas of conducting based on CNFs stacking; and (**d**) Raman analysis which represents a comparison of different elements of the AFM image for the pure CNFs, the pure SF and nanocomposite containing SF with CNFs density equal to 0.4%.

In Figure 5a, the interdependence between the frequency and impedance of analyzed samples is presented. According to Figure 5a, the addition of the CNFs concentration in the SF sample decreases impedance and consequently increases the electrical conductivity of the material when compared with the conductivity of the pure SF sample. Therefore, with the increase in the CNFs concentration (0.1% to 0.5%) the electrical conductivities of the SF sponges are also improved. Actually, the larger concentration of the CNFs amplifies the number of charge carriers in the SF polymer matrix. This is confirmed in the result also obtained by Li et al. in [34], which observed that hybrid fibers composed of the SF and carbon nanotubes (CNTs) experience an abrupt increase in conductivity up to the 638.9 Sm^−1^ when exceeding the percolation threshold of CNTs, which is CNTs >35 wt%. In the study presented in this work the concentration of the CNFs is low, and the CNFs cannot provide a continuous conducting network that can exhibit frequency-independent behavior. However, the conducting nature of CNFs helps to reduce the impedance significantly from 17.9 kΩ to 8.5 kΩ at 20 MHz (Figure 5a).

The AFM conducting images (obtained with Kelvin probe) presented in Figure 5b,c show the combinations of interchanged conducting and piezoelectric zones. These combinations help in improving piezo response in its transfer from the interior of the composite to its surface. Figure 5c represents magnified parts of Figure 5b. Magnification shows that the CNFs have high contrast due to their conductivity. The pure SF zone is the darkest in Figure 5c, while the SF with CNF depression equal to 0.4% shows brighter image contrast.

The Raman spectra presented in Figure 5d for the high, medium and low conducting zones confirm the impact of the CNFs positioning in the SF matrix. The peaks at 1083 cm^−1^, 1266 cm^−1^ and 1665 cm^−1^ in Figure 5d arise from the Silk II (beta sheet conformation). The weak absorption at the level equal to 1109 cm^−1^ implies the existence of a small amount of Silk I (shaped as a random coil or a helical which is also confirmed in [26]). The other peak presented in Figure 5d at 1444 cm^−1^ (due to methylene (CH_2_) scissoring) is independent of silk fibroin and it is also confirmed in [34]. The peaks at 1300 cm^−1^ and 1592 cm^−1^ are related to the CNFs that resemble the D and G bands of carbon atoms, respectively, which is also confirmed in [35].

From these characterizations, the AFM image of the analyzed sample presented in Figure 5b,c and Raman analysis of the sample presented in Figure 5d are used to explain the enhanced piezoelectric effect with the CNFs inclusion in the SF matrix. The positioning of the CNFs in the matrix is regulated such that the piezoelectric SF lies between the conducting CNFs. This enables the staking effect, which leads to an enhanced piezoelectric effect on the composite sponge. Ma et al. in [36] also observed such alignments of conducting CNTs between SF matrix, which lead to improved signal responses through a change in the capacitance of the system.

Along with the increase in piezoelectric Silk II content (characterized by the increase in CNFs), this arrangement of CNFs remarkably enhanced the piezoelectric response of the SF by improving beta (β) phase crystallinity (methanol treatment) for the case of adding CNT/graphene [36]. In CNF polymer composites the tunneling effect is the key mechanism of electrical conduction. Hence, the electrical conductivity of the CNF polymer composites is affected by the thickness of the polymer layer of the CNF surfaces, and this thickness depends on the implemented surface treatment method and the polymer type. This electrical conduction enhances the conductivity when compared to samples with other combinations of CNF densities.

### 4.3. Compression Analysis of Sponges

**Figure 6:**
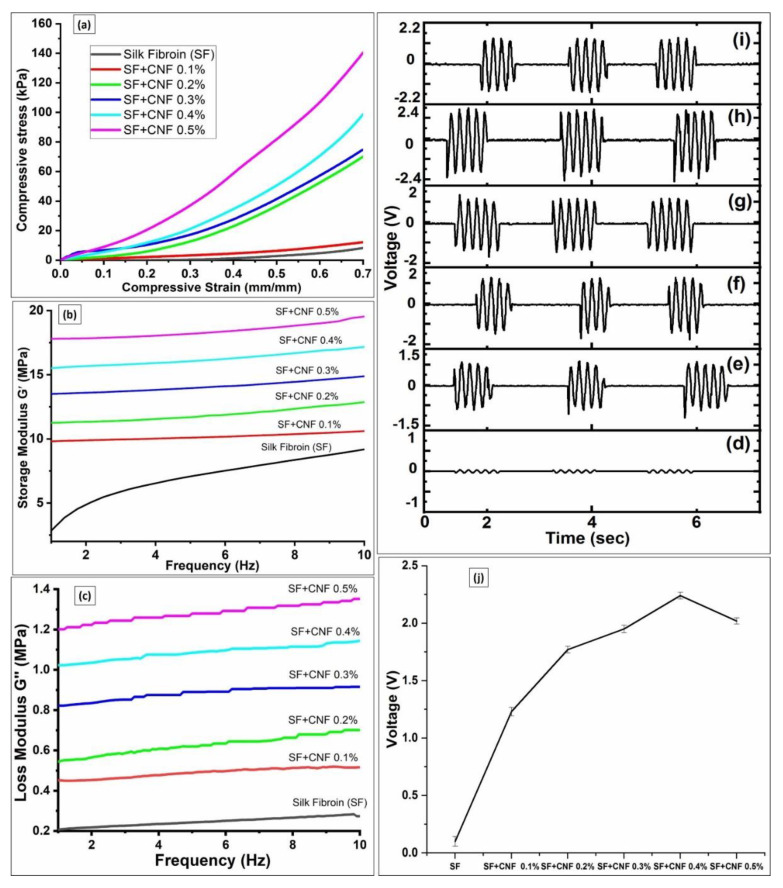
(**a**) Stress vs. strain analyses for SF samples with different CNF concentrations; (**b**) Storage modules vs. frequency analysis for the SF samples with different CNF concentrations; (**c**) Loss modulus vs. frequency analyses for SF samples with different CNF concentrations; Piezoelectric voltage generated for the samples composed of: (**d**) the pure SF, (**e**) the SF with CNFs density equal to 0.1%; (**f**) the SF with CNFs density equal to 0.2%; (**g**) the SF with CNFs density equal to 0.3%; (**h**) the SF with CNFs density equal to 0.4%; (**i**) the SF with CNFs density equal to 0.5%; (**j**) Peak average values of generated piezoelectric voltage for SF samples with different CNF concentrations.

Figure 6a shows the stress vs. strain graphs of developed SF with CNF samples. Results presented in Figure 6a shows that the addition of the CNFs improves the mechanical properties. All of the samples containing CNFs showed remarkable compressive strength improvements [29]. The SF sample with CNF density equal to 0.5% attains maximum compressive strength ranging up to 140 ± 1.2 kPa at 70% strain. The pure SF sample has a yield strength of 6 ± 0.22 kPa, which indicates that the addition of CNFs in the silk significantly enhances its strength by more than 20 times. The CNFs have successfully reinforced SF sponge structures, making the structure more robust to mechanical strains. Adding CNFs also contributes to the reduction in the porosity of the material, which also helps to improve the compressive strength of the sponges.

### 4.4. Dynamic Mechanical Analysis

The DMA was performed further to analyze the mechanical strength and behavior of the SF with CNFs samples. The storage modulus (G′) and loss modulus (G″) were studied and the results of analyses are shown in Figure 6b,c. It had been observed that the addition of the CNFs concentration increases the silk storage modules’ strength (Figure 6b). During the experiments the temperature was kept at 37 °C, which is near to the human body temperature. All the samples were tested at frequencies ranging between 1 Hz and 10 Hz. For the increase in CNFs densities the storage modulus has shown at 1 Hz frequency an increase from 1 MPa to 18 MPa. However, with the increase in frequency the pure SF sample has not shown a very significant increase in storage modulus, which at the 10 Hz frequency equals 9.1 ± 0.04 MPa. For analyses performed at a frequency of 10 Hz, the SF samples having CNFs density equal to the 0.4% and 0.5% obtain a significant increase in storage models’ strange capabilities equal to 17.1 ± 0.031 MPa and 19.5 ± 0.06 MPa, respectively. Hence, the SF with the CNFs density of 0.5% exhibits the best storage modulus compared with other samples (Figure 6b). In the loss modulus study presented in Figure 6c, the increase in loss modulus has similar trends to those of storage modules for all analyzed SF samples with different CNF densities. While the pure SF obtain the loss module strength of 0.3 ± 0.012 MPa, the SF with CNF density equal to 0.5% has shown 1.3 ± 0.024 MPa loss module strength at 10 Hz. Based on the obtained results it can be seen that adding CNFs contributes to achieving the higher flexibility and elastic property of the material, while the viscosity property of the material also increases between the SF samples having different CNFs ranging from 0.1% to 0.5% (*w*/*v*). Therefore, in both storage and loss modulus the mechanical properties were significantly increased due to the increment of the CNFs concentration.

### 4.5. Piezoresponse under Cyclic Loading

An in-house developed cam setup was used to analyze the voltage responses from the prepared samples. Figure 6d–i show the piezoelectric voltage response of the SF samples with different CNF densities under cyclic loading conditions, monitored and recorded in the oscilloscope. Table 1 shows the average measured piezoelectric voltage obtained for the 100 testing cycles. This executed large number of testing cycles is selected in order to have statistical relevance of the obtained results.

According to obtained results presented in Figure 6d–i, the addition of the CNFs to the SF matrix improves its piezoelectric performance due to the conducting nature of the CNFs. With the increase in CNFs, concentration voltage improvement from 0.8 V to 2.2 V was achieved (Table 1). The highest piezoelectric voltage response has been achieved for the SF sample with the CNFs density equal to 0.4% (*w*/*v*), which reached 2.2 ± 0.12 V at the low current equal to 0.1 µA. Compared to results obtained for the piezoelectric voltage response of the PVDF samples in [28], obtained piezoelectric voltage response is better. Moreover, results presented in Figure 6h,i and Table 1 show that the concentrations of the CNFs densities higher than 0.4% (e.g., SF with CNF density equal to 0.5% with sample thickness equal to 3.92 ± 0.12 mm) provide no further benefit in terms of piezoelectric voltage (2.01 ± 0.20 V) response.

Since the principle of collecting piezoelectric signals is based on the transfer of the charge generated due to pressure to the attached electrodes in order to obtain the piezoelectric power from the sensor, the CNFs help in this regard by facilitating the flow of generated charges towards the electrode. In Figure 6j, peak average values of generated piezoelectric voltage with corresponding deviations for the SF samples having different CNF densities are presented. According to the results presented in Figure 6j the highest conductivity is obtained for the SF with CNFs densities equal to 0.4% and 0.5%. This is a consequence of the high distribution of the CNFs in the SFs. The other SF samples have a lower generation of piezoelectric voltages due to lower CNFs densities added into the SF (Figure 6j). However, the higher CNF concentrations (above 0.4%) result in a better conducting pathway, which may cause induction that results in short-circuit conditions in the SF samples. This is the main reason why the increase in the CNFs densities above 0.4% does not bring further improvements in piezoelectric voltage generation (Figure 6j).

Additionally, the obtained results presented in Table 1 show that the SF with CNF densities equal to 0.4% and 0.5% had thickness recoveries equal to 99.2 ± 0.4% and 99.3 ± 0.2%, respectively. The pure SF without CNFs had a slightly inferior recovery of 97 ± 0.1%. Other nanocomposite SF sponges having different CNF densities recovered thicknesses in the range from 97 ± 0.5% to 99 ± 0.2%. The highest sensitivity for the SF with the CNF density equal to 0.4% is 2.95 V/cm^2^. Therefore, obtained results show that after 100 test cycles all the tested SF samples demonstrated remarkable usage repeatability and recoverability, with no damage or deformation.

### 4.6. Continuous Cyclic Load and Voltage Response Analyses

**Figure 7:**
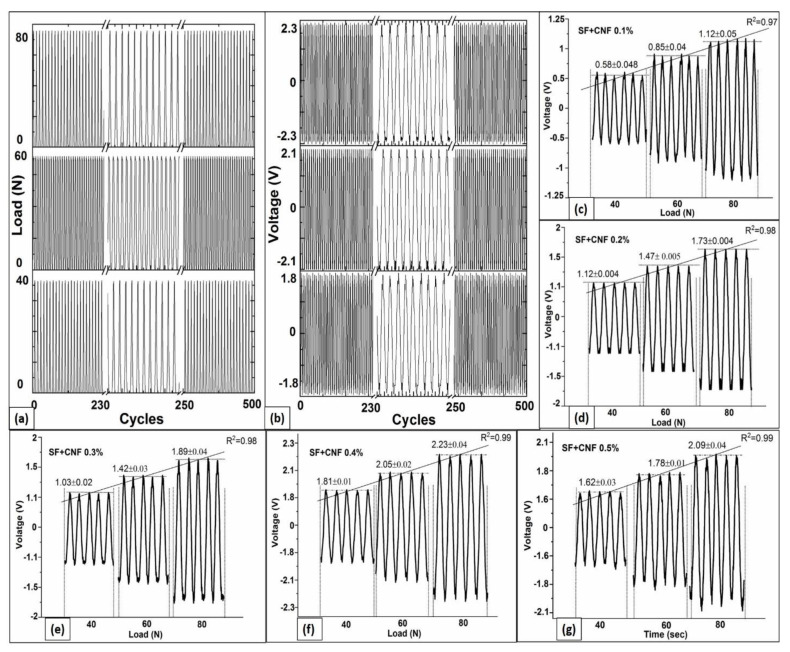
(**a**) Load Response analyses for the SF with CNF density equal to 0.4%; (**b**) Voltage Response analyses for the SF with CNF density equal to 0.4%; Piezoelectric voltage response on three different load levels of the SF sensor with CNF densities equal to: (**c**) 0.1%; (**d**) 0.2%; (**e**) 0.3%; (**f**) 0.4% and (**g**) 0.5%.

The SF samples with CNF nanocomposites were analyzed through continuous compression tests repeated in 500 cycles, for which piezoelectric voltage responses have been recorded in parallel. This executed large number of testing cycles is selected in order to have statistical relevance of the obtained results. Figure 7a shows the waveforms of the generated cyclic load (measured using motorized tension/compression test stand force gauge) and Figure 7b shows corresponding voltage responses (measured using DSO) for the analyzed SF sample with CNF densities equal to 0.4%. According to the results presented in Figure 7a,b, the high responsiveness in terms of generated piezoelectric voltage induced by continuous compression is achieved for the SF-based sensor having the CNFs density equal to 0.4%.

Table 2 summarizes the voltage response at three different load levels (equal to 40 N, 60 N and 80 N) for 500 compression test cycles, and indicates results obtained for the compression recovery of the material after load tests performed for load equal to 80 N. According to the results presented in Table 2, the pure SF did not show a significant piezoelectric voltage response (0.1 ± 0.01 V) for all three loading compressions. Although studies with pure SF show the existence of piezoelectric responses due to the processing techniques related to the improvement of Silk II content and their alignments [22], in this study neither of such techniques has been adopted. This is because the goal of this study was to precisely assesses the influence of the CNF on SF-based sensor piezoelectricity and implementation of any of such processing techniques will influence SF piezoelectricity response. However, according to the results presented in Figure 7c–g, for SF with different CNF densities (ranging from 0.1% to 0.5%) a significant change in piezoelectric voltage is generated for every one of the analyzed load levels. In addition, according to results presented in Figure 7c–g, the increase in the CNF in the SF structure up to 0.4% of CNF density improves the level of piezoelectric voltage response as well as mechanical properties, which according to Table 2 exhibit high load recovery.

Additionally, Figure 7c–g highlights changes in instantaneous piezoelectric voltage responses and Table 2 presents the piezoelectric voltage variations for three different levels of compression loads (40 N, 60 N and 80 N). According to results presented in Figure 7c–g, there exists a linear correlation between the generated instantaneous piezoelectric voltage and increased load levels for any of the analyzed SF samples containing different CNF densities. The highest linearity is observed for the SF samples having CNF densities equal to 0.4% and 0.5% (both showing statistical measures of fit R^2^ equal to 0.99). This observed level of high linearity is a consequence of the CNF presence, of which one of the characteristics is mechanical strength. Therefore, these results show that combining the SF and CNF has a vital role in the increment of mechanical strength of the SF surface. Furthermore, results show that the addition of CNFs in SF improves tensile strength, and the larger CNF percentages in the SF to CNF aspect ratios can improve the mechanical strength. As indicated in [37], such larger aspect ratios reduce the possibility of sensors cracking.

According to the results presented in Table 2, the total voltage response for the SF with CNF density equal to 0.5% is lower than those for 0.4% density (Table 2). Since the recovery rate of the SF with CNF density equal to 0.4% is just slightly lower (99.1 ± 0.15%) than those for 0.5% density (99.4 ± 0.18%), the degradation of mechanical characteristics is not significant, while piezoelectricity response of SF with CNF density equal to 0.4% is larger. For that reason, the SF with CNFs density equal to 0.4% is further used for the performance analyses of the developed SF-based sensor in real wireless wearable applications.

### 4.7. Testing of Various Human Motions

**Figure 8:**
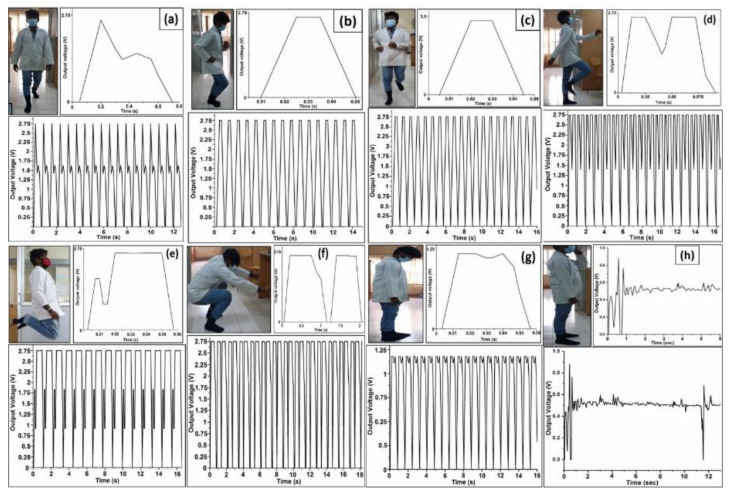
The piezoelectric voltage response of the SF-based sensor with CNF densities equal to 0.4%, analyzed for various human motions which include: (**a**) walking; (**b**) jogging; (**c**) running; (**d**) marching; (**e**) jumping; (**f**) squatting; (**g**) tapping and (**h**) standing [Video S1: movement analyses].

In this section, the results of real testing of a developed SF-based sensor with CNFs density equal to 0.4% are presented and discussed. The results were obtained for the real application scenario characterized by different human body motions (Figure 8). Three volunteers differing in age, gender, weight and height (as indicated in Table 3) were selected to perform different human motion activities. These motion activities include walking, jogging, running, marching, jumping, squatting, tapping and standing with the attached SF-based sensor implanted in socks (Figure 8). One SF-based sensor with a CNFs density equal to 0.4% was stitched in the toe area of the sockets. When toe area pressure caused by human movement is applied, the generated piezoelectric voltage is measured. For every step of the foot strike, the volunteer’s toe area pressure was calculated for the corresponding portion of the toe area. From the developed sensor, the generated output voltage response was wirelessly recorded to the LabView software.

The measured results are presented in Figure 8 for each of the analyzed human motion activities [Video S1: movement analyses], where an upper figure of each subfigure in Figure 8 shows in detail one measuring sample for the short time duration, while the bottom figure of each subfigure shows measuring results for the larger number of measuring samples obtained during a longer measuring period lasting up to 10 s or more. According to the measured results presented in Figure 8, different human movements generate piezoelectric voltage through the application of pressure on the SF-based sensor attached to the toe area of the socks. This output voltage and corresponding current response are the results of the piezoelectric characteristics of the material used for the development of the SF-based sensors. Table 3 shows the variations in the piezoelectric voltage generated by the SF-based sensor for each of the analyzed human body movements. According to Figure 8 and Table 3, piezoelectric voltage response may differ due to the volunteer’s body weight, the toe area size and shape and the way of performing specific movements by the volunteer. According to results presented in Table 3, among different volunteer characteristics, the body weight seems to have the highest impact on generated piezoelectric voltage. More specifically, the volunteers with larger weights generate a higher piezoelectric voltage in comparison with the volunteers with lower body weights, for each of the analyzed movements. According to the obtained results, it is clearly visible that the developed SF-based sensor can generate piezoelectric voltages for any of the analyzed movements and human body types and can be used in real practical implementations.

### 4.8. Cell Viability

**Figure 9:**
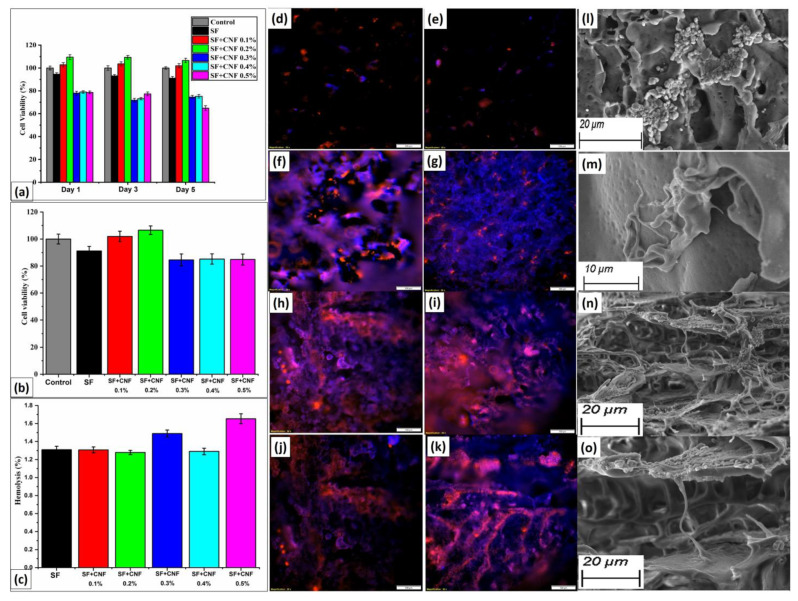
(**a**) Percentage of skin cells viability for samples with different CNF densities in the SF; (**b**) Percentage of blood cells viability for samples with different CNF densities in the SF; (**c**) Percentage of hemolysis for samples with different CNF densities in the SF; Cytotoxicity studies of: (**d**) the pure SF; (**e**) the SF with CNF density equal to 0.1%; (**f**) the SF with CNF density equal to 0.2%; (**g**) the SF with CNF density equal to 0.3%; (**h**) the SF with CNF density equal to 0.4%; (**i**) the SF with CNF density equal to 0.5%; (**j**) the untapped SF with CNF density equal to 0.4%; (**k**) the tapped SF with CNF density equal to 0.4%; (**l**) untapped pure SF sample with cells; (**m**) tapped pure SF sample with cells; (**n**) untapped SF with 0.4% sample with cells; and (**o**) tapped SF with 0.4% sample with cells.

The last analyses presented in this work have been dedicated to the cytotoxic analyses of the SF and SF with CNF samples. The cytotoxic effect of the SF sample and SF with CNF samples was tested with human dermal fibroblast (HADF) cells using the MTT assay. Results of analyses have been presented in Figure 9 for cell viability, hemolysis and cytotoxicity mapping.

According to the results presented in Figure 9a, the SF and SF with CNF samples attain more than 70% of cell viability after Day 1 (incubation time 24 h) and Day 3 (incubation time 72 h). The cell viability above 70% remains on Day 5 (incubation time 120 h) for all SF with CNF samples, except the SF with CNF samples density equal to 0.5% (Figure 9a). Obtained results confirm that the proposed sensor based on SF with CNF retains a high percentage of cell viability for longer periods.

Because of such SF properties, particularly its tunable insolubility and very mild processing, SF could be an excellent candidate for immobilizing and preserving biological and nonbiological molecules. Such cell viability can be considered cell supportive and non-toxic, which is also confirmed in [29]. This cytotoxicity study shows that the developed samples can be utilized for biomedical applications based on skin contact.

Further, the SF and SF with CNF samples were tested for their cytotoxicity with THP-1 human monocyte-like cells using an MTT assay. The results are similar to fibroblast cells, having more than 90% of cell viability after 48 h of incubation time, as shown in Figure 9b. According to [29], such cell viability can be also considered as cell supportive and non-toxic to blood cells. This cytotoxicity study shows that the developed samples may be utilized for implementation in biomedical applications.

### 4.9. In Vitro Hemolysis Assay

Another analysis performed was in vitro hemolysis of the SF samples with different CNF densities. Figure 9c depicts the results of the in vitro hemolysis assay study performed for the various combination of SF samples and the SF samples with different CNF densities. The data in the histogram revealed that the hemolysis rate of the materials ranges from approximately 1.2% to 1.7%, which is consistent with the international standard for hemolysis of medical biomaterials (which defines hemolysis ratio to be less than 5%). This means that the cell proliferation rate increased as the number of days increased. The findings suggested that SF with CNF sponges could be used for in vivo medical field applications.

### 4.10. Visualization of Cytotoxicity Analyses

Final analyses represent a visualization of the cytotoxicity maps. In Figure 9d–i, the cytotoxicity studies of the pure SF and the SF with CNF density ranging from 0.1% to 0.5% have been presented for the unit resolution of cytotoxicity maps equal to 100 μm. Figure 9j shows the visualization of the cytotoxicity maps for untapped SF with CNF density equal to 0.4%, and in Figure 9k for the tapped SF with CNF density equal to 0.4% is presented. The maps of the cytotoxicity analyses for the untapped and tapped pure SF sample with cells have been presented in Figure 9l,m, respectively. Finally, the maps of the cytotoxicity analyses for the untapped and tapped SF with CNF density equal to 0.4% with cells have been presented in Figure 9n,o, respectively. According to the presented cytotoxicity maps the SF-based material, as a biodegradable product, was tested for cytotoxicity and found to be cell-safe and nontoxic. Therefore, the developed SF-based sensor has biocompatible nature and can be implemented on humans.

## 5. Discussion

With a focus on various human motion sensing, analyses presented in this work provided an update on the most recent research results in the field of implementing smart wearable SF-based sensors. Results of analyses show that the development of new SF with CNF sensors increases the capabilities of biosensing technologies such as e-skins, e-textiles and e-healthcare, which have great potential to be fully practically implemented for biosensing in the future.

Furthermore, age-related health problems include a variety of biological changes, the advent of illnesses that are frequently chronic, and a reduction in cognitive functions. As an example, the combination of intrinsic and extrinsic fall risk factors makes fall prediction a difficult problem to solve. Age, fall history, mobility issues, sleep issues and neurological diseases are all intrinsic variables. Sleep disorders as extrinsic fall risk are quite prevalent in elderly persons and consequently, irregular gait was present in 35% of non-institutionalized seniors. Therefore, it is obvious that when people age and develop diseases affecting their locomotor system their entire gait pattern becomes altered. However, gait monitoring systems have so far been developed in laboratory settings under controlled conditions without tests performed on frequent fallers or elderly test subjects. Therefore, future research should concentrate on long-term investigations of fall detection and prediction systems on a variety of people, including those who fall frequently, aging adults, kids, sports persons, those with neurological problems and defense personnel. Research should be performed in real environments during daily indoor and outdoor human activities.

Future research and commercialization efforts related to the development of effective and precise gait monitoring systems must also overcome many obstacles. Materials, technology and the testing environment are among the most important challenges. Concerning challenges related to materials, polymer nanocomposites, for example, are widely utilized in sensor design; however, they exhibit very low long-term stability and such materials are easily oxidized. The challenge of long-term stability and oxidation is the main obstacle that should be overcome if long-term implementations of such sensors want to be achieved.

Also, the relevance of gait sensing approaches strongly depends on the number of human participants involved in testing. According to the reported research works, 56% of the articles discuss gait research with up to 20 participants, while only 20% of the articles discuss studies with 51 or more participants. Most of the research works that were reported under the topics related to “gait and fall”, “physical activity recognition”, “rehabilitation” and “stress and sleep” including this work had up to 20 human persons participating in tests. We consider that such a relatively low participant count is a drawback, and for that reason future investigations with a larger number of test subjects can bring more reliable biosensors performance results.

## 6. Conclusions

In this paper, a novel SF-based sensor with different CNF densities has been developed for possible medical applications as a human wearable sensing device. The development process of the SF-based sensor with different CNF densities has been described in detail.

The results of analyses show that adding CNFs in SF material improves the piezoelectric characteristics of the material, and that the developed SF-based sensor with CNF density equal to 0.4% obtains the highest piezoelectric voltage response equal to 2.95 ± 0.03 V. Moreover, the results of the analyses show that the mechanical response of the developed SF-based sensor corresponds to more than 99% thickness recovery after being subjected to different cyclic compressive loads equal to 40 N, 60 N and 80 N.

The developed piezoelectric SF-based sensors were further tested in real medical applications on humans for detecting generated piezoelectric voltage in versatile body motions like walking, jogging, running, marching, jumping, squatting, tapping and standing. Obtained results show that the proposed SF-based sensor has an appropriate piezoelectric sensitivity to every analyzed body movement type, and that the proposed SF-based sensor can be applied in real medical applications as a biomedical sensing device.

Moreover, performed cytotoxicity analyses of the developed SF-based sensor show that the developed sensor has a non-toxic and biocompatible nature, which opens the avenue for the sensor to in vivo biomedical applications realized through the implementation of wearable health monitoring devices. In our future research activities, the sensors will be implemented for the gait monitoring studies for a larger number of normal and abnormal patients in hospitals. The final product will be commercialized for implementation in medical health monitoring applications.

## Figures and Tables

**Table 1:** The voltage response of SF with different CNFs densities.

Material	Piezoelectricity (V)	Resistance (kΩ)	Thickness (mm)
SF	0.1 ± 0.01	293	3.07 ± 0.14
SF with CNFs densities equal to 0.1%	1.2 ± 0.18	164	3.25 ± 0.19
SF with CNFs densities equal to 0.2%	1.87 ± 0.22	133	3.42 ± 0.13
SF with CNFs densities equal to 0.3%	1.92 ± 0.32	119	3.63 ± 0.15
SF with CNFs densities equal to 0.4%	2.2 ± 0.12	88	3.75 ± 0.11
SF with CNFs densities equal to 0.5%	2.01 ± 0.20	57	3.92 ± 0.12

**Table 2:** Generated piezoelectric voltage and compressive recovery of SF nanocomposite samples after 500 testing cycles.

Samples	Piezovoltage (V) after 500 Test Cycles			Compressive Recovery Rate after 500 Test Cycles for 80 N Load (%)
	Load			
	40 N	60 N	80 N	
SF	0.1 ± 0.01	0.1 ± 0.01	0.1 ± 0.01	95.1 ± 0.24
SF with CNF densities equal to 0.1%	0.45 ± 0.22	0.91 ± 0.18	1.23 ± 0.17	97.2 ± 0.26
SF with CNF densities equal to 0.2%	1.03 ± 0.20	1.42 ± 0.22	1.87 ± 0.12	97.7 ± 0.18
SF with CNF densities equal to 0.3%	1.05 ± 0.12	1.43 ± 0.21	1.91 ± 0.26	98.6 ± 0.19
SF with CNF densities equal to 0.4%	1.77 ± 0.18	2.03 ± 0.19	2.25 ± 0.24	99.1 ± 0.15
SF with CNF densities equal to 0.5%	1.54 ± 0.15	1.72 ± 0.20	2.01 ± 0.19	99.4 ± 0.18

**Table 3:** Piezoelectric voltage recordings obtained on different volunteers for versatile body movements.

Volunteer Characteristics	Volunteer 1	Volunteer 2	Volunteer 3
**Age**	26	31	28
**Weight (kg)**	55	89	71
**Height (cm)**	148	159	151
**Gender**	Male	Male	Female
**Motion movements**	**Voltage (V)**		
Walking	2.34 ± 0.11	2.51 ± 0.12	2.41 ± 0.11
Jogging	2.21 ± 0.19	2.42 ± 0.21	2.39 ± 0.21
Running	2.42 ± 0.05	2.72 ± 0.14	2.71 ± 0.12
Marching	2.12 ± 0.09	2.45 ± 0.04	2.41 ± 0.14
Jumping	2.65 ± 0.12	2.95 ± 0.03	2.87 ± 0.12
Squatting	2.62 ± 0.16	2.88 ± 0.09	2.91 ± 0.15
Tapping	1.11 ± 0.09	1.86 ± 0.09	2.10 ± 0.19
Standing	2.55 ± 0.08	2.87 ± 0.09	2.71 ± 0.01

## Data Availability

Not applicable.

## Supplementary Materials

The following supporting information can be downloaded at: https://www.mdpi.com/article/10.3390/s23031373/s1, Video S1: movement analyses.

## Abbreviations

SF	Silk Fibroin
CNF	Carbon Nanofiber
SEM	Scanning Electron Microscope
FTIR	Fourier Transform Infrared Spectroscopy
PZT	Lead Zirconate Titanate
BaTiO_3_	Barium Titanate
PVDF	Polyvinylidene Fluoride
PLA	Polylactic Acid
MTT	(3-(4,5-dimethyl thiazolyl-2)-2, 5-diphenyltetrazolium bromide)
SKM	Scanning Kelvin Probe Microscopy
AFM	Atomic Force Microscope
DI	Deionized Water
DMA	Dynamic Mechanical Analysis
SRF	Solid Rectangular Fixture
PEG	Polyethylene Glycol
